# Efficacy of a fluralaner-based ectoparasiticide for the control of otodectic and sarcoptic mange in naturally infested dogs, evaluated in randomized, double-blind clinical studies

**DOI:** 10.1186/s13071-026-07279-3

**Published:** 2026-02-22

**Authors:** Breno Cayeiro Cruz, Marina Belucci Teixeira, Gessica Ariane de Melo Cruz, Juliana Aparecida do Carmo Emidio Moreira da Silva, Milenni Garcia Michels, Carol Della Nina Pistoni, Marcus Antonio Martins Buso, Ferdinando Nielsen de Almeida, Igor Renan Honorato Gatto

**Affiliations:** 1 Ourofino Saúde Animal Ltda., Cravinhos, São Paulo, Brazil; 2 Graduate Program, School of Veterinary Medicine and Animal Science, São Paulo State University (Universidade Estadual Paulista “Júlio de Mesquita Filho”), Botucatu, São Paulo, Brazil

**Keywords:** Acaricide, Isoxazoline, Mites, *Otodectes cynotis*, *Sarcoptes scabiei*, Efficacy, Treatment

## Abstract

**Background:**

One of the most relevant parasitic mites is *Sarcoptes scabiei*, which causes sarcoptic mange, commonly known as scabies. This condition is highly contagious and leads to intense itching and skin lesions in dogs. *Otodectes cynotis*, the “ear mite,” is immensely relevant as well, being the primary cause of external otitis in domestic carnivores, causing severe ear itching. The treatment and control of both these forms of mange rely on the use of antiparasitic compounds, such as fluralaner, which acts by paralyzing and killing arthropods. This study aimed to determine the acaricidal efficacy of WellPet™ (Ourofino Saúde Animal Ltda.), a new commercial palatable tablet based on fluralaner, at a dose range of 10–22.5 mg/kg, against *S. scabiei* and *O. cynotis* mites parasitizing naturally infested domestic dogs.

**Methods:**

Two clinical studies evaluate the acaricide efficacy against each mite species in naturally infested animals. Each study utilizes 14 animals each, divided into a Control Group (*n* = 7), treated with a reference product on the basis of sarolaner (a palatable tablet based on sarolaner, 2.0–4.0 mg/kg—Zoetis Indústria de Produtos Veterinários Ltda.), and a treated group (*n* = 7) treated with WellPet™ (a palatable tablet based on fluralaner, 10–22.5 mg/kg—Ourofino Saúde Animal Ltda.). Acaricidal efficacy is assessed in both trials through mite counts and evaluation of lesions resulting from the diseases.

**Results:**

Against *Sarcoptes scabiei*, WellPet™ provided a significant reduction in mite counts from day 14 (*p* = 0.022) onward, reaching 100% efficacy by day 28 without need for retreatment. The control group showed significant reduction from day 7 (*p* = 0.045) onward, but required retreatment to reach 100% efficacy by day 44. In the *Otodectes cynotis* trial, the WellPet™ group showed significant reduction starting from day 3 (*p* = 0.036), with 94.1% efficacy by day 14 and 100% from day 21 onward. The control group showed significant reduction starting from day 7 (*p* = 0.022) and reached 100% efficacy by day 14.

**Conclusions:**

The WellPet™ product, a novel ectoparasiticide based on fluralaner, proved to be highly effective in the treatment of both sarcoptic and otodectic mange, with results similar to those of sarolaner, the only isoxazoline, at the time of these trials, registered in Brazil for controlling these mites. The main advantage observed for WellPet™ is the ability to achieve complete cure of sarcoptic mange with a single dose, thereby simplifying disease management. For otodectic mange, both products were highly effective. Further trials must still be conducted to corroborate these initial findings, but in summary, data presented here indicates that WellPet™ is an effective and practical treatment option for sarcoptic and otodectic mange in dogs with a single administration.

**Graphical Abstract:**

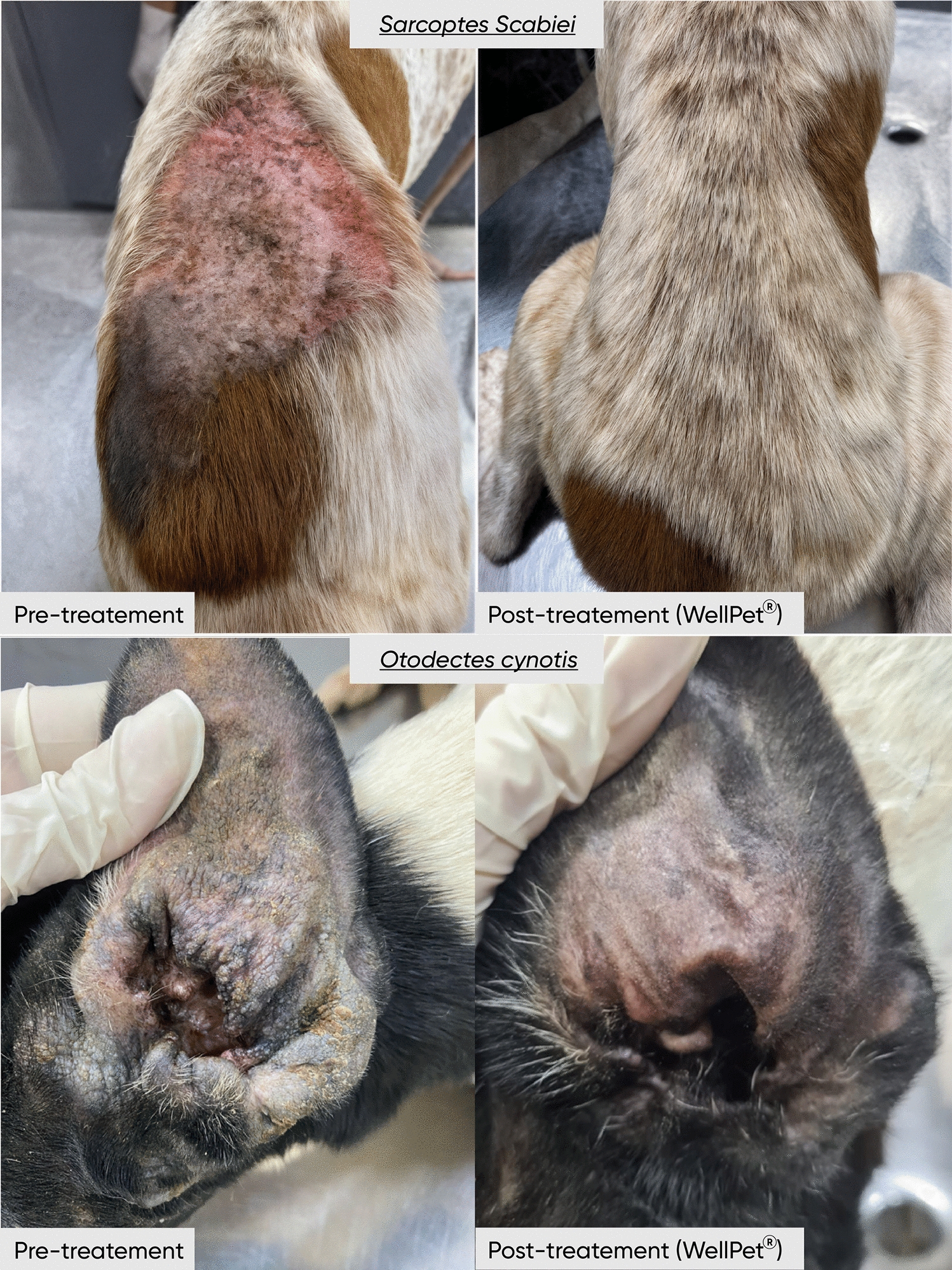

**Supplementary Information:**

The online version contains supplementary material available at 10.1186/s13071-026-07279-3.

## Background

Mite infestations rank among the most prevalent and challenging dermatopathies encountered in small animal practice. Canine sarcoptic mange (*Sarcoptes scabiei* var. *canis*) and otodectic mange in dogs (*Otodectes cynotis*) together represent a significant burden to animal health [1–4].

Canine sarcoptic mange is a cosmopolitan, highly contagious, nonseasonal dermatopathy that affects dogs of all breeds, ages, and sexes [2, 5]. Its pathogenesis involves female mites burrowing into the stratum corneum, where they tunnel, feed, and deposit eggs and fecal pellets. In addition to mechanical irritation, the mites secrete allergenic substances that trigger an intensely pruritic hypersensitivity reaction in sensitized dogs [5, 6]. Clinically, the disease is characterized by severe pruritus, papules, erythema, alopecia, crusts, and excoriations, with early predilection sites including the extremities and ventral body regions; the dorsal midline being often spared [1, 7]. Definitive parasitological diagnosis is achieved via positive skin scrapings, considered the diagnostic gold standard [1, 3]. Prevalence of parasitic dermatitis in dogs may reach up to 35.4% in some regions [8].

In parallel, otodectic mange (otoacariasis), caused by *O. cynotis*, is among the most common causes of external otitis in dogs and cats [1, 9]. These mites inhabit the surfaces of the horizontal and vertical ear canals [1]. High prevalence has been documented in regional studies in Brazil [10]. Within the ear canal, the mite induces accumulation of mild-to-heavy, brown-dark, ceruminous or crusty exudate [1, 6]. Chronic inflammation may lead to severe structural changes in the external ear canal, including glandular and epithelial hyperplasia, ductal dilatation, and hyperkeratosis [9, 11]. These changes predispose to increased cerumen production and elevated local pH, facilitating secondary bacterial (e.g., *Staphylococcus* spp., *Pseudomonas*, *Proteus*) and yeast (*Malassezia* spp.) infections [1, 9, 11]. Infested animals typically present intense ear pruritus; head-shaking and scratching, which results in secondary alopecia and excoriations around the head and ears; and repeated trauma, which may cause aural hematomas [1]. Both conditions are of zoonotic and sanitary relevance. Sarcoptic mange is well documented for its zoonotic potential, while otodectic mange has been occasionally linked to external otitis in humans, although such cases are rare and typically involve close contact with infected animals [2, 12].

Therapeutic management of these mite infestations has evolved, with an emphasis on systemic acaricides. Among systemic options, the isoxazoline class (including fluralaner, sarolaner, and lotilaner) has emerged as a potent group of parasiticides [3, 13–16].

At nanomolar concentrations, fluralaner acts as a potent blocker of ligand-gated chloride channels, mainly gamma-aminobutyric acid (GABA)-gated and glutamate (GLU)-gated chloride channels, the former being exclusive to arthropods. This action prevents the influx of chloride ions into postsynaptic tissue, leading to depolarization, paralysis, and death of the parasite [17, 18]. In addition to its potency and selectivity, fluralaner has a unique molecular structure compared with other known chloride channel blockers, targeting the resistant to dieldrin (RDL) subunit of GABA receptors at a distinct binding site, which differs from other chloride channel inhibitors [18].

Fluralaner is widely recognized as an effective alternative for controlling both *S. scabiei* and *O. cynotis* in a variety of mammals. At dosages of 25–56 mg/kg, it has been effective in dogs (both mites), cats (only *O. cynotis*), rabbits, maned wolves, raccoon dogs, bare-nosed wombats, koalas, and alpacas (only *S. scabiei* in all these mammals) [13]. However, a limited number of studies have evaluated its safety profile, and none have assessed long-term effects. In dogs, oral administration at doses of 25–56 mg/kg has been associated with gastrointestinal signs (vomiting, reduced appetite, diarrhea, flatulence) and general clinical signs (lethargy, polydipsia) [13]. Rare but severe adverse events, such as pemphigus foliaceus or neurological manifestations (impaired gait, oral dysphagia, muscle tremors, generalized ataxia), have also been reported at this superior dose range [13].

Multiple recent studies show similar efficacies among available acaricides [13]. Yet, evaluating formulations that combine high performance against both *S. scabiei* and *O. cynotis* remains highly relevant, especially new products with an increased safety profile, as such products may simplify treatment protocols and improve compliance in clinical practice.

Therefore, the aim of this study was to evaluate the acaricidal efficacy of WellPet™ (Ourofino Saúde Animal Ltda.), a new palatable fluralaner-based oral formulation administered at doses ranging from 10 to 22.5 mg/kg, in naturally infested dogs with *Sarcoptes scabiei* var. *canis* or *Otodectes cynotis*.

## Methods

Because of the biological differences between the two mite species, two independent studies were conducted, both being positive-controlled, double-blind, randomized, multicenter clinical trials. Each study was based on protocols specifically designed to address the unique characteristics of the target parasite. Both trials complied with international good clinical practice guidelines (VICH GL9) and national animal welfare regulations, and were previously approved by the relevant Animal Use Ethics Committee.

Each trial included 14 animals divided equally between an investigational veterinary product (IVP) treated group (*n* = 7) and a positive control group (*n* = 7). Eligible animals were over 12 months of age, weighed more than 2 kg, were microchipped for identification, and free of health conditions unrelated to *S. scabiei* or *O. cynotis* infestations. All animals were diagnosed as being naturally infested with at least five live mites [19], and enrolled after owner consent via a signed informed consent form. Animals showing clinical illness, undergoing treatment, having received any medication other than vaccines or anthelmintics within 30 days before study start, or exhibiting excessive aggressiveness were excluded. During the trials, bathing, walking, contact with other animals, or administration of any unapproved medication, including antiparasitics, were prohibited.

Randomization followed a preestablished alternating allocation sequence to ensure balanced group distribution. On day 0 (D0), animals in the IVP treated group received WellPet™ (Ourofino Saúde Animal Ltda.) orally at the label-recommended minimum dose of 10 mg/kg of fluralaner. Animals in the positive control group received an oral reference antiparasitic containing sarolaner, at the minimum recommended dose of 2 mg/kg (Table 1). Due to the fixed-dose format of the commercial tablets, individual doses were administered according to the manufacturer’s weight-based recommendations, which could result in slight variations from the theoretical minimum dose.

**Table 1:** Description of the doses administered to the animals included in the studies

Animal ID (microchip)	Group	Animal weight (kg) on day −1	Amount of PVI and control product administered (mg) on day 0	Dose (mg/kg)
Study 1—*S. scabiei*				
1273	TG	15.45	200.00	12.94
445	TG	14.55	200.00	13.75
447	TG	14.30	200.00	13.99
1310	TG	7.70	100.00	12.99
5096	TG	23.10	400.00	17.32
5141	TG	25.50	400.00	15.69
446	TG	9.85	100.00	10.15
9344	CG	11.00	40.00	3.64
9164	CG	14.10	40.00	2.84
1309	CG	16.10	40.00	2.48
9408	CG	15.80	40.00	2.53
1249	CG	13.80	40.00	2.90
658	CG	7.60	20.00	2.63
9165	CG	10.10	40.00	3.96
Study 2—*O. cynotis*				
3065	TG	12.65	200.00	15.81
8929	TG	17.95	200.00	11.14
5127	TG	16.50	200.00	12.12
5067	TG	21.10	400.00	18.96
5070	TG	20.80	400.00	19.23
4849	TG	11.45	200.00	17.47
5200	TG	18.50	200.00	10.81
3044	CG	17.80	40.00	2.25
3056	CG	11.25	40.00	3.56
3063	CG	40.00	80.00	2.00
5065	CG	16.00	40.00	2.50
4844	CG	16.70	40.00	2.40
4846	CG	25.15	80.00	3.18
8503	CG	20.80	80.00	3.85

*ID* identification

*TG* treated group

*CG* control group

In the *Sarcoptes scabiei* study, infestation confirmation and post-treatment assessments were based on skin scrapings from five affected areas (≥ 2.5 cm^2^ each) per animal, collected after scraping until capillary bleeding was observed. Scrapings were examined microscopically (10× magnification) by a blinded veterinarian, and mite counts corresponded to the total number of live mites found across all areas. Dermatological lesion severity was also assessed in two areas per animal based on pruritus, erythema, papules, excoriations, crusts, alopecia, and pyoderma.

Dermatological assessment of lesion severity was conducted prior to sample collection in two areas on the basis of the following parameters: pruritus, erythema, papules, excoriations, crusts, alopecia, and pyoderma.

On days +3, +7, +14, +21, and +28 after treatment, clinical evaluations, acaricide efficacy evaluations, and dermatological evaluations were performed. If mites were still present on day 28 (D +28)—i.e., if the final post-treatment assessment revealed at least one live mite—a second treatment was administered on day 30 (D +30), in accordance with the monthly dosing schedule recommended for the reference product, and proven as safe for both WellPet™, though its recommended posology is for use every 45 days. Following the second treatment, if and when needed, new evaluations were conducted on days +33, +37, +44, +51, and +58.

In the trial focused on *Otodectes cynotis*, infestation was confirmed by otoscopic examination, complemented by material collection from both ear canals and external/middle ear regions (≈1 cm^2^) using a sterile swab. Collected samples were microscopically examined for mites and eggs, and counts from both sides were combined as the final value per animal. Lesions were evaluated for signs associated with otodectic mange, such as head shaking, pruritus, trauma, erythema, ulceration, alopecia, and debris.

Both studies were conducted at a contracted research organization by independent veterinarians not affiliated to the study sponsor. At each site, activities were distributed between a nonblinded dispenser, responsible for assigning and administering treatments, but who did not participate in clinical evaluations, and a blinded examiner, who performed all treatment-related observations.

The main criterion for evaluating the product’s efficacy in each trial was the parasitological cure rate, calculated as the proportion of dogs that showed negative results for *S. scabiei* or *O. cynotis*. Efficacy was calculated, using the adapted Abbott’s formula, at each post-treatment date [20, 21]:

$$\begin{aligned} {\text{Efficacy Percentage }}\left( \% \right) & = \left\{ {\left( {{\text{mean live mite counts in animals on D}} - {1}} \right)} \right. \\ & \quad - \left( {{\text{mean live mite counts on D}} + {\mathrm{X}}} \right) \\ & \quad \left. { \div \left( {{\text{mean live mite counts in animals on D}} - {1}} \right)} \right\} \times {1}00 \\ \end{aligned}$$

At this formula, D + X refers to each of the post-treatment counts. For a product to be considered an adequate acaricide, its calculated efficacy must be at least above 90% [22].

All data generated in each study were statistically evaluated using Student’s *t*-test (parametric) or Mann–Whitney (non-parametric) for comparisons between groups and between treatment times. Intragroup comparisons were also performed using the paired *t*-test (parametric) or Wilcoxon test (nonparametric), depending on data distribution. Statistical tests were performed with the aid of both the SPSS program, version 22, developed by IBM (2013) and the Minitab® Statistical Software (© 2025 Minitab, LLC.). Statistical significance would be considered when the *p*-value was less than 5% (*p* < 0.05).

### Determination of adequate sample sizes (*N*):

The Experimental Power Graphing Program spreadsheet was used in the study design to determine the test power and the appropriate number of repetitions [23]. Mean mite counts obtained in previous studies by the same research group (11 ± 4 mites per animal) were confirmed by literature data [24, 25]. These values met the normality assumption without transformation, and the program tab for data with a constant coefficient of variation (CV) was used, since standard deviations varied proportionally to the means. This approach, considered conservative, generally requires larger sample sizes to achieve the desired power. A significance level of 0.05 and a test power of 0.8 were adopted. On the basis of simulations of different hypotheses and sample sizes, seven animals per group were estimated as sufficient to detect reductions of 50% or more in mite counts with confidence greater than 95%. This number was therefore considered adequate to ensure statistical robustness while complying with the principle of using the minimum number of animals necessary.

Additionally, the sample size of seven animals per group is consistent with previously published efficacy studies for mange treatments in dogs, which frequently utilize samples of similar or slightly larger size to demonstrate significant results. For instance, studies [11, 24, 26] employed a comparable number of animals per group to evaluate the efficacy of different treatments for parasitic infestations.

In conclusion, though further trials might be needed to corroborate the initial findings of these two studies, taking in to account the solid method for determining the sample sizes, existing support with the same numbers of animals being used in similar trials, and direct application of the 3R concept, it is safe to assume that a larger *N* was not necessary for the results obtained and the conclusions achieved to be trustworthy.

## Results

### Study 1—Sarcoptes scabiei

In total, 14 animals were included in the study, comprising 42.9% male (6/14) and 57.1% female (8/14), with a mean age of 4.7 years (animals aged 1–9 years).

Regarding the clinical condition of animals, the occurrence of abnormal clinical signs was observed. However, these were only changes resulting from verminosis or stress of the animals at the time of clinical evaluations. Some animals presented grade 2 (mild) dehydration (3–4 s), regarding capillary refill time (CRT). However, by the end of the study, these returned to normality. Additionally, all animals exhibited normal behavior throughout the whole trial.

**Figure 1:**
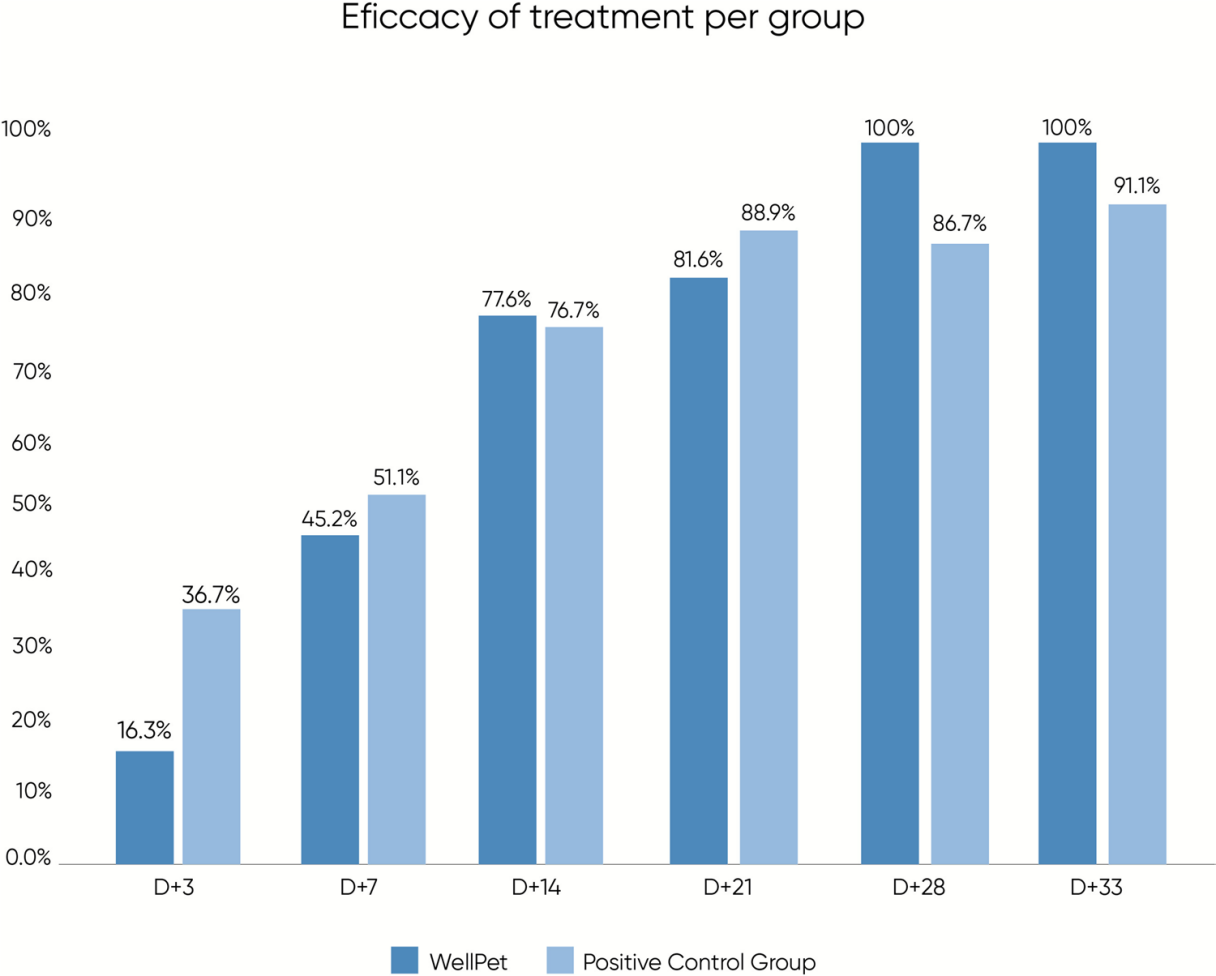
Efficacy of treatment against Sarcoptes scabiei with WellPet^TM^ or Reference Product (Positive Control Group), during the experimental period

After the first negative result for each animal’s mite counts, confirmation was obtained through additional skin scrapings, performed at weekly day intervals, totaling three consecutive negative results. At the end of the 30-day evaluation period, after the first treatments, two animals in the control group still tested positive for mites, requiring a second sarolaner administration. No animals treated with WellPet™ needed a second fluralaner administration. Figure 1 presents the mean mite counts for each group at each experimental timepoint.

Statistical analysis of the total mite counts per animal (Table 2) revealed distinct intragroup trends. The control group showed a statistically significant reduction in mite counts from day 7 (*p* = 0.045) onward, while the WellPet™-treated group reached statistical significance starting from day 14 (*p* = 0.022). Nonetheless, WellPet™ achieved full 100% efficacy by day 28, without the need for retreatment. The control group, however, required retreatment for two animals to reach 100% efficacy by day 44. Intergroup comparison showed no statistically significant differences (*p* > 0.05). These results confirm WellPet™’s effectiveness, surpassing the minimum threshold of 90% efficacy [22], completely controlling *S. scabiei* infestations 4 weeks after a single administration.

**Table 2:** Scabicidal efficacy results (mite count) against *S. scabiei* and intragroup and intergroup statistical analyses of treated groups

Timepoint (day)	Group 1—WellPet™ (single oral dose of fluralaner, 10–22.5 mg/kg)			Group 2—Control (single oral dose of sarolaner, repeated after 30 days if necessary)				Intergroup *p*-value
	Mean ± SD (N)	Efficacy percentages	Intragroup (versus day −1) *p*-value	Mean ± SD (N)	Efficacy percentages	Intragroup (versus day −1) *p*-value		Group comparison
D-1	14.00 ± 6.24 (*n* = 7)	–	–	12.86 ± 3.63 (*n* = 7)	–	–		*p* = *0.683*
D +3	11.71 ± 8.06 (*n* = 7)	16.3%	*p* = *0.152*	8.86 ± 6.01 (*n* = 7)	36.7%	*p* = *0.103*		*p* = *0.467*
D +7	7.57 ± 8.10 (*n* = 7)	45.9%	*p* = 0.082*	6.29 ± 6.87 (*n* = 7)	51.1%	*p* = 0.045*		*p* = *0.754*
D +14	3.14 ± 4.71 (*n* = 7)	77.5%	*p* = 0.022*	3.00 ± 3.06 (*n* = 7)	76.7%	*p* = 0.003*		*p* = *0.947*
D +21	0.29 ± 0.76 (*n* = 7)	81.6%	*p* = 0.022*	1.43 ± 2.44 (*n* = 7)	88.9%	*p* = 0.022*		*p* = *0.259*
D +28	0.00 ± 0.00 (*n* = 7)	100%	–^a^	1.71 ± 2.98 (*n* = 7)	86.7%	*p* = 0.022*		*p* = *0.154*
D +33	0.00 ± 0.00 (*n* = 3)	100%	–^a^	2.75 ± 3.20 (*n* = 4)	91.1%	*p* = 0.01*		*p* = *0.149*
D +44	–^a^	100%	–^a^	0.00 ± 0.00 (*n* = 2)	100%	–^a^		–^a^
D +51	–^a^	–^a^	–^a^	0.00 ± 0.00 (*n* = 2)	100%	–^a^		–^a^
D +58	–^a^	–^a^	–^a^	0.00 ± 0.00 (*n* = 2)	–*	–^a^		–^a^

^*^ Statistically significant

^a^Not enough data to assess normality; statistical analysis not applicable

In the evaluation of skin lesions resulting from sarcoptic mange, no statistically significant differences were observed between the groups for any of the analyzed variables throughout the study.

**Figure 2:**
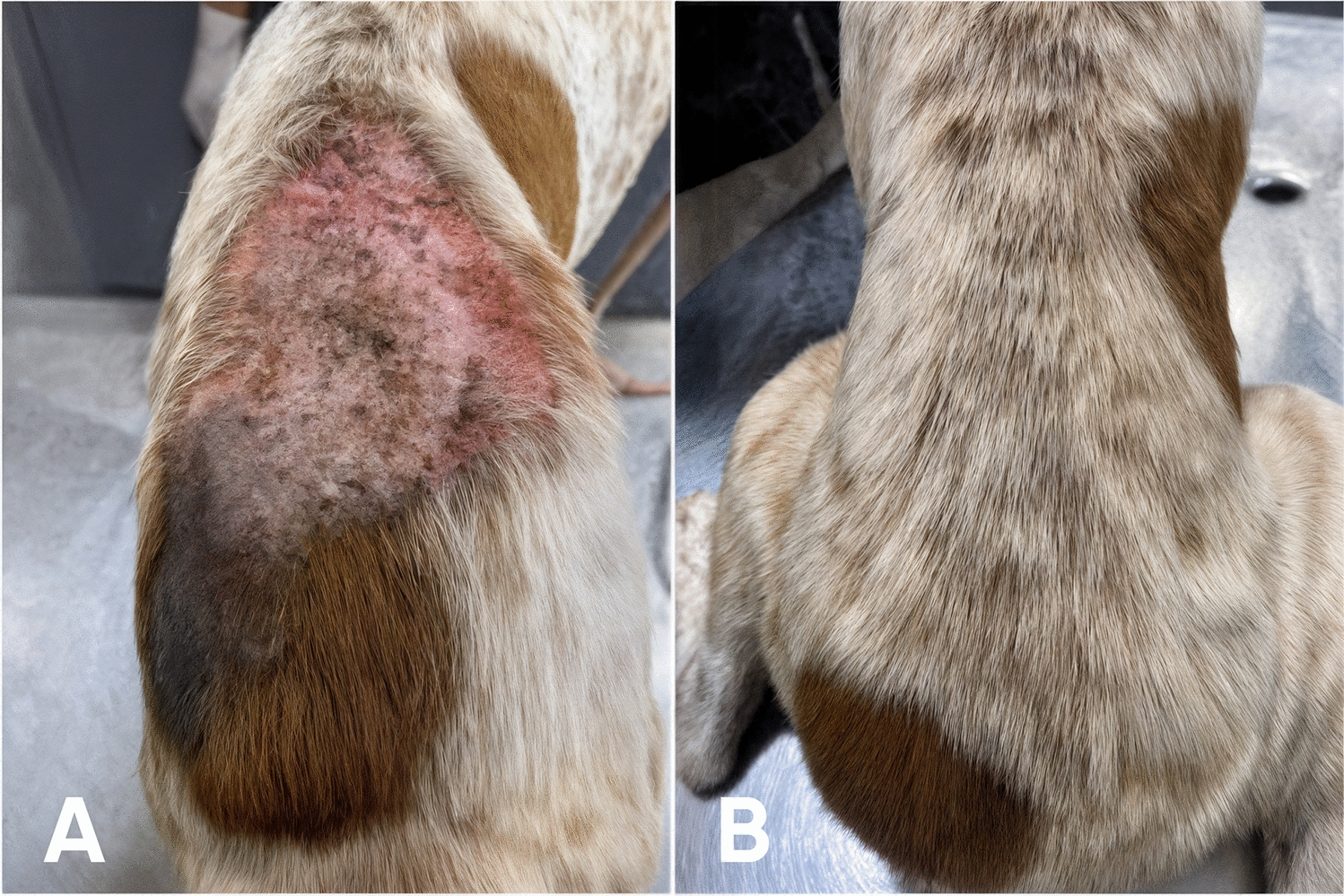
Representative images showing skin excoriations in an animal treated with WellPet^TM^ at baseline (**A**) and the same animal with complete remission of the lesions at D+28 (**B**)

**Figure 3:**
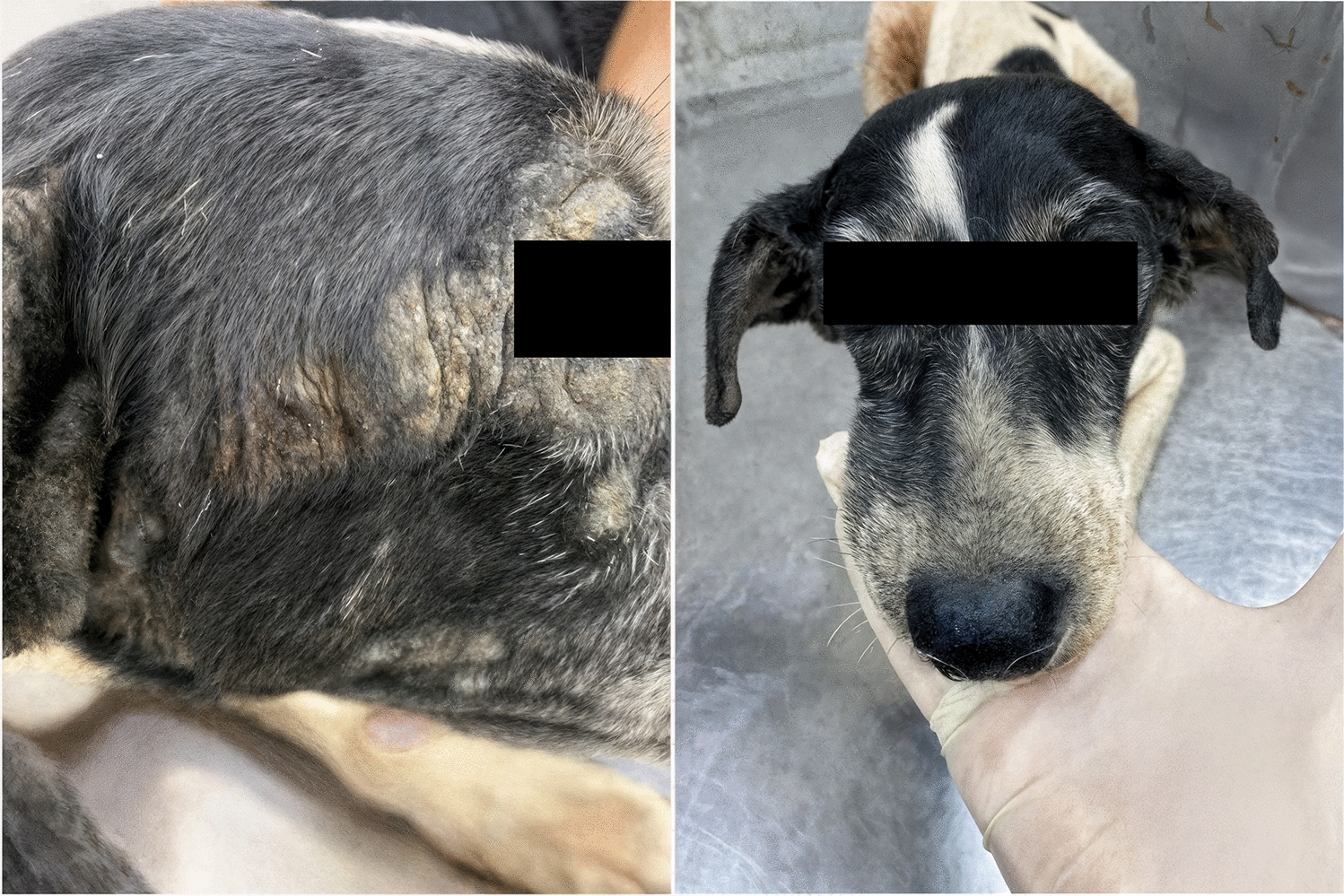
Representative images showing crusted lesions in an animal treat with WellPet^TM^ at baseline (**A**) and the same animal with complete remission of the lesions at D+28 (**B**)

From D +28 onward, pruritus was completely absent in animals treated with WellPet™. In contrast, animals in the control group only achieved total absence of pruritus by D +37. Similarly, erythema resolved entirely in the treated group by D +14 and remained absent until the end of the study, whereas in the control group, complete resolution was only observed starting from D +28. Excoriations disappeared entirely in the treated group as early as D +14 (Fig. 2), while in the control group, this occurred only by D +21. In both cases, erythema was not detectable anymore even before total elimination of *S. scabiei* infestations. Regarding crusted lesions (Fig. 3), total resolution was observed on D +14 in the treated group and even earlier in the control group, on D +7. Finally, alopecia was completely resolved in the treated group by D +37, while in the control group, full recovery was only observed at the end of the study, on D +58. For alopecia, as expected, complete resolution only happened after total elimination of mite infestations.

### Study 2—Otodectes cynotis

Another 14 animals were included in this study, comprising once again 42.9% male (6/14) and 57.1% female (8/14), with a mean age of 4.7 years (animals aged 1–10 years). Regarding the clinical picture, only the occurrence of abnormal clinical signs related to a positive diagnosis for *O. cynotis* were observed.

**Figure 4:**
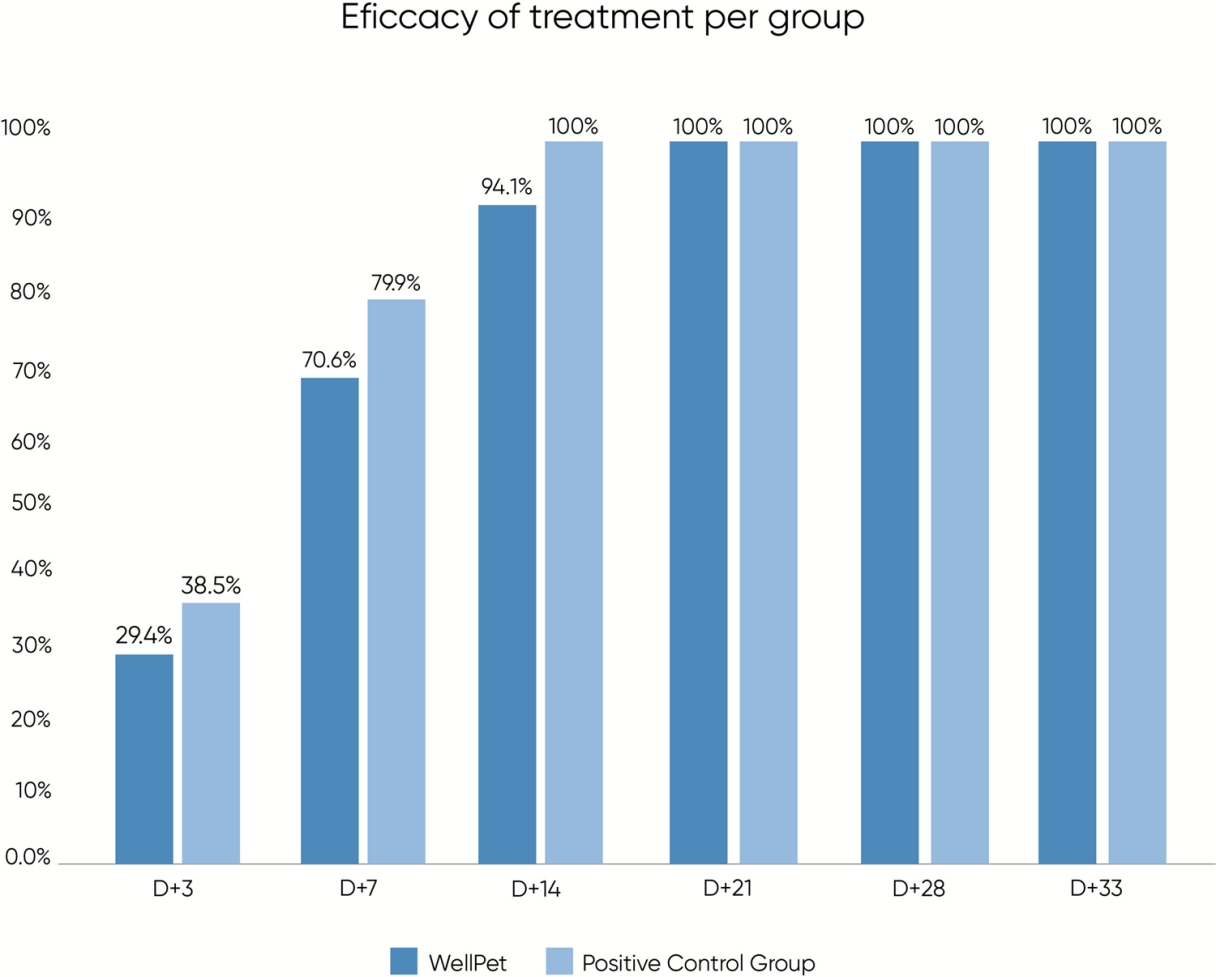
Efficacy of treatment against Otodectes cynotis with WellPet^TM^ or Reference Product (Positive Control Group), during the experimental period

At the end of the 30 days of evaluation, after treatment of animals with single dosages of each product, all animals in both groups showed negative results for mites, confirmed in at least three consecutive negative collections. Therefore, no second treatment was required for any animal. A small number of animals from each group still had mites detected on D +14, and only began to show complete negativity from D +21—specifically, two dogs in the treated group and three in the control group. For these animals, an additional evaluation on D +33 was necessary to complete the three required negative assessments. WellPet™ demonstrated 94.1% efficacy as early as day 14, reaching 100% from day 21. Figure 4 shows the means of mite counts for each group at each experimental timepoint.

**Figure 5:**
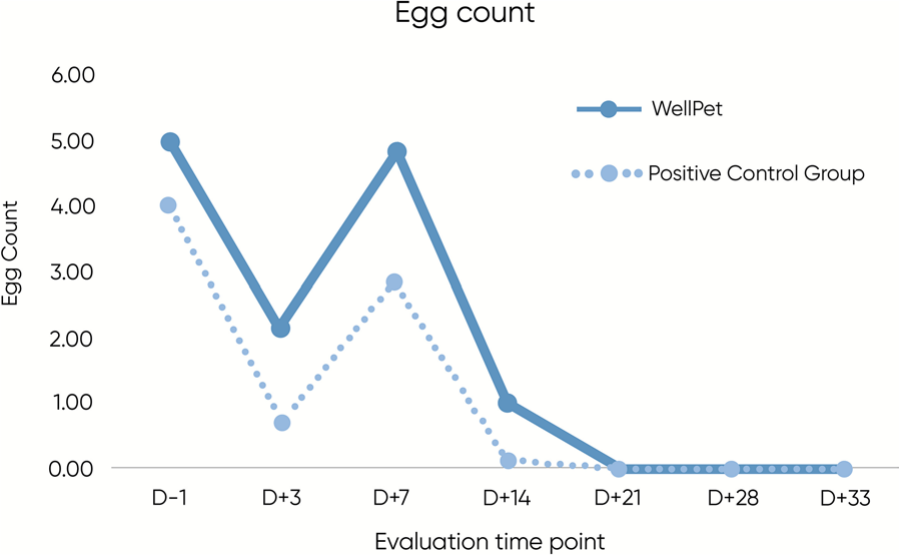
Mean mite egg counts (Otodectes cynotis) of animals medicated with WellPet^TM^ (TG) or reference product (CG), during the experimental period

Upon analyzing the mite count data, which represents the final average count from the right and left ears, statistical results are summarized in Table 3. In the intragroup analysis, the treated group demonstrated a statistically significant reduction in mite counts compared with baseline as early as day 3 post-treatment (*p* = 0.036). The control group, in turn, achieved a statistically significant reduction from day 7 (*p* = 0.022) onward. Though the control group achieved 100% efficacy first, by day 14, in the intergroup analysis, no statistically significant differences were observed between the groups (*p* > 0.05). Furthermore, egg count data, presented in Fig. 5, also showed no statistically significant differences between the groups. As this parameter served only as a complementary factor for the efficacy assessment, and not as a primary outcome variable, intragroup analysis was not performed.

**Table 3:** Scabicidal efficacy results (mite count) against *O. cynotis* and intragroup and intergroup statistical analyses of treated groups

Timepoint (day)	Group 1—WellPet™ (single oral dose of fluralaner, 10–22.5 mg/kg)			Group 2–Control (single oral dose of sarolaner, repeated after 30 days if necessary)			Intergroup *p*-value
	Mean ± SD (N)	Efficacy percentages (%)	Intragroup (versus day −1) *p*-value	Mean ± SD (N)	Efficacy percentages (%)	Intragroup (versus day −1) *p*-value	Group comparison
D −1	17.14 ± 6.74 (*n* = 7)	–	–	13.00 ± 8.10 (*n* = 7)	–	–	*p* = *0.319*
D +3	12.43 ± 7.02 (*n* = 7)	29.4	*p* = 0.036*	7.71 ± 2.93 (*n* = 7)	38.5	*p* = *0.053*	*p* = *0.127*
D +7	4.38 ± 3.53 (*n* = 7)	70.6	*p* = 0.001*	2.86 ± 1.21 (*n* = 7)	76.9	*p* = 0.022*	*p* = *0.182*
D +14	1 ± 1.41 (*n* = 7)	94.1	*p* = 0.022*	0.29 ± 0.49 (*n* = 7)	100	*p* = 0.022*	*p* = *0.23*
D +21	0.00 ± 0.00 (*n* = 7)	100	–^a^	0.00 ± 0.00 (*n* = 7)	100	–^a^	–^a^
D +28	0.00 ± 0.00 (*n* = 7)	100	–^a^	0.00 ± 0.00 (*n* = 7)	100	–^a^	–^a^
D +33	0.00 ± 0.00 (*n* = 3)	100	–^a^	0.00 ± 0.00 (*n* = 2)	100	–^a^	–^a^

^*^ Statistically significant

^a^Not enough data to assess normality; statistical analysis not applicable

In the evaluation of skin lesions resulting from otodectic mange, there was no statistical difference for any of the analyzed variables. However, it is worth noting that, despite the resolution of other dermatological signs by the end of the study, some animals in both groups still exhibited mild alopecia. The persistence of alopecia in these animals is likely due to the fact that hair regrowth is a dermatological process that requires a longer recovery period.

**Figure 6:**
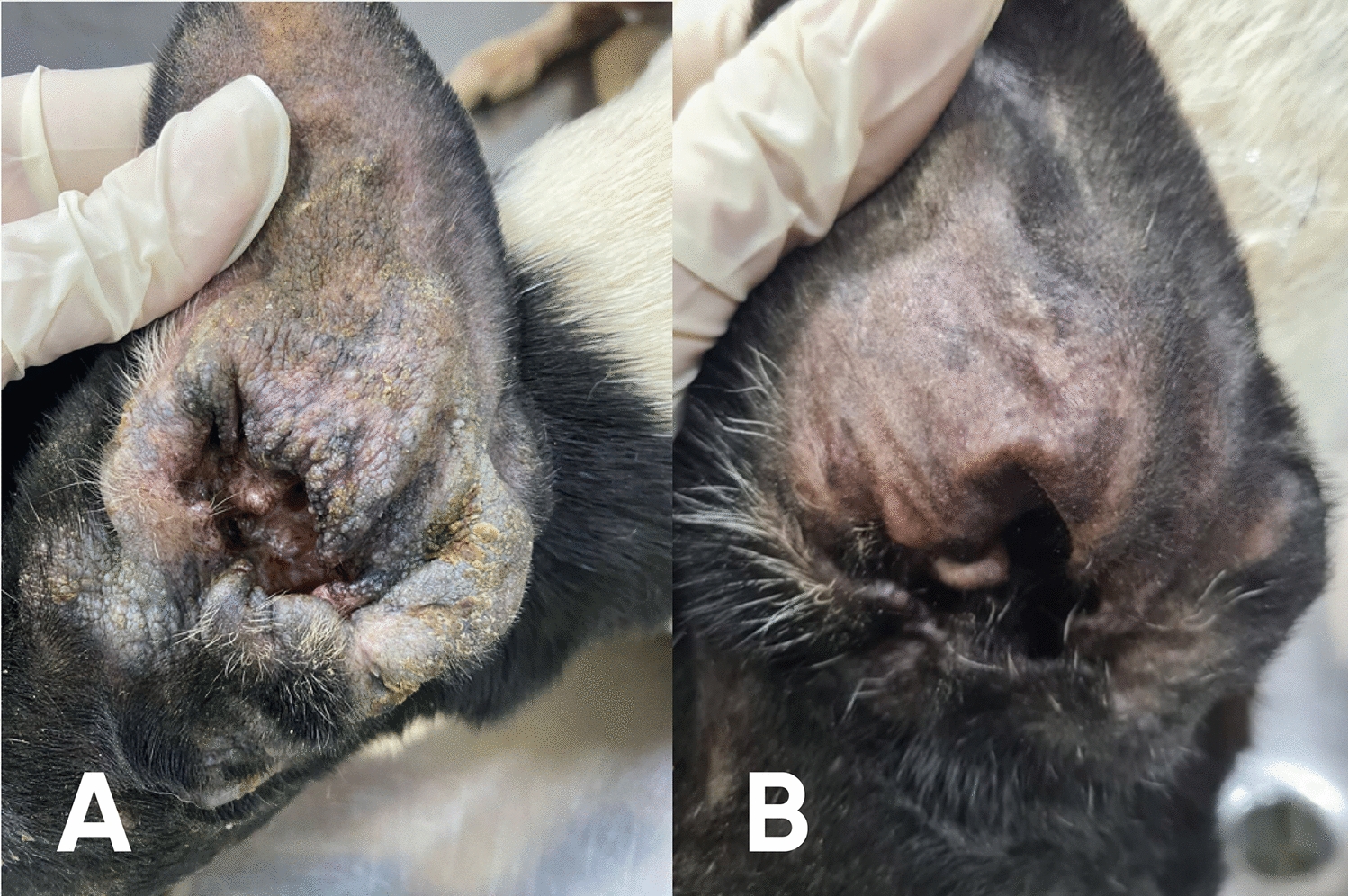
Representative images showing an animal treat with WellPet^TM^ at baseline **(A)** and the same animal with full remission of clinical signs at D+28 **(B)**

It is also noteworthy that, from D +7, erythema was already completely absent in the group treated with WellPet™, whereas in the control group, this was only observed from D +14. Both treatments achieved 100% acaricidal efficacy with a single application, as evidenced by the complete elimination of mites confirmed through consecutive weekly counts, along with full remission of clinical signs associated with otodectic mange (Fig. 6).

## Discussion

WellPet™ demonstrated high efficacy, achieving complete control in all animals (100%) 28 days after a single administration, with a dose of 10–22.5 mg/kg of fluralaner. Furthermore, complete remission of associated clinical signs, such as pyoderma, pruritus, erythema, excoriations, crusts, and alopecia, was observed, with no animal in the study requiring readministration until the end of the follow-up period (D +58). Although no statistically significant differences were observed compared with the control group, the latter only achieved complete efficacy after the second administration.

These findings are consistent with available literature. Previous studies have reported similar efficacy against *Sarcoptes scabiei* [24], demonstrating that a single dose of fluralaner, administered orally or topically, resulted in 100% reduction in *S. scabiei* counts after 4 weeks. Similarly, another study [25] reported that more than 90% of dogs were mite free on day 28, with 100% efficacy on days 56 and 84, lasting up to 12 weeks. In that same study, the control group treated with sarolaner required two administrations (days 0 and 28) to achieve complete mite elimination on days 56 and 84—a treatment approach that was also necessary in the present study to ensure full resolution in some animals.

Similar outcomes have been documented in studies evaluating the use of sarolaner in combination [16, 27], also with two monthly doses, achieving 99.2% reduction on day 60 in a smaller-scale study and complete parasitological clearance in the same period in a larger-scale study. The need for multiple administrations for sarolaner, as evidenced in this study and consulted literature, reinforce a practical advantage of WellPet™ regarding the simplicity of the therapeutic protocol, which may improve treatment adherence by owners and clinicians.

In the case of otodectic mange, WellPet™ demonstrated 94.1% efficacy as early as day 14, reaching 100% from day 21 onward. Additionally, complete remission of clinical signs associated with the disease, such as head shaking, pruritus, trauma or alopecia, erythema, ulceration, and debris, was also verified.

These results suggest a faster clinical response under the conditions evaluated in relation to the results observed with afoxolaner [26], whose efficacy was 98.5% (geometric mean) and 99.4% (arithmetic mean) only on day 28, reinforcing the potential of fluralaner as an effective alternative in the control of canine otodectic mange. The clinical response observed in this study is also corroborated by published material from other authors [11], which reported a 99.8% reduction in *O. cynotis* counts on day 28 after a single oral dose of fluralaner, with the absence of visible mites on otoscopy as early as day 14 or 28 in most cases.

The present study emphasizes several advantages of the evaluated formulation. First, oral administration ensures systemic drug distribution, allowing the active compound to reach parasites located in deeper dermal layers (sarcoptic mange) or in anatomically difficult sites (otodectic mange) [1], and avoids issues related to topical administration such as accidental removal by grooming or bathing. Second, the use of a lower fluralaner dose (10–22.5 mg/kg) provides efficacy comparable to higher doses reported in the literature (25–56 mg/kg), while potentially reducing the risk of adverse effects [13]. This lower-dose, single-administration approach simplifies treatment protocols, minimizes systemic exposure, and may improve owner compliance and therapeutic outcomes.

The sample size used in this study was determined through a rigorous a priori power analysis, as detailed in the Methodology section, employing a conservative rationale on the basis of the coefficient of variation (CV) and aiming for a test power greater than 95% to detect efficacies ≥ 50%. The choice of a minimal number of animals reflects an ethical commitment and compliance with the principle of reduction in animal experimentation, seeking statistical robustness with the fewest experimental units necessary. Although the sample size of *n* = 7 was statistically justified to meet the study’s primary objectives, the low number naturally imposes limitations on the generalizability of the findings. Therefore, similar studies should be conducted with larger sample sizes and under diverse experimental conditions to confirm the consistency of the results and enhance the external validity.

## Conclusions

The results confirm the high efficacy of WellPet™ (fluralaner administered orally, at a single dose of 10–22.5 mg/kg) in the treatment of both sarcoptic and otodectic mange, showing comparable performance to sarolaner, the only isoxazoline registered in Brazil for those same indications at the time of the study. The single-dose regimen allowed complete parasitological clearance of sarcoptic mange and rapid clinical improvement in otodectic mange, supporting its use as an effective and convenient oral therapeutic option.

## Supplementary Information

Additional file1 (PDF 285 kb)

## Data Availability

Data supporting the main conclusions of this study are included in the manuscript.
