# Supplementary material for: Efficacy of a fluralaner-based ectoparasiticide for the control of otodectic and sarcoptic mange in naturally infested dogs, evaluated in randomized, double-blind clinical studies
Source: Parasit Vectors. 2026 Feb 22;19:107. doi: 10.1186/s13071-026-07279-3 (PMC12973857; doi:10.1186/s13071-026-07279-3)
Supplement: Supplementary file 1 — Additional file1 (PDF 285 kb) [file 13071_2026_7279_MOESM1_ESM.pdf]

Table 1. Power of the test calculated for each hypothesis (percentage of difference between mite counts and distinct potential number of repetitions evaluated), considering a confidence level of 0.05 (α = 0.05, i.e., 95% confidence interval) and 0.8 (1-β = 80%) as the minimum acceptable power for the test.

Percent difference	Sample size (n per group)									
	2	3	4	5	6	7	8	9	10	11
-50%	0.075	0.281	0.506	0.666	0.775	<u>0.848</u>	<u>0.898</u>	<u>0.932</u>	<u>0.955</u>	<u>0.970</u>
-55%	0.084	0.362	0.618	0.771	<u>0.862</u>	<u>0.917</u>	<u>0.950</u>	<u>0.970</u>	<u>0.982</u>	<u>0.989</u>
-60%	0.095	0.457	0.721	<u>0.853</u>	<u>0.921</u>	<u>0.957</u>	<u>0.977</u>	<u>0.988</u>	<u>0.993</u>	<u>0.996</u>
-65%	0.109	0.557	<u>0.806</u>	<u>0.910</u>	<u>0.957</u>	<u>0.979</u>	<u>0.990</u>	<u>0.995</u>	<u>0.998</u>	<u>0.999</u>
-70%	0.126	0.653	<u>0.870</u>	<u>0.947</u>	<u>0.978</u>	<u>0.990</u>	<u>0.996</u>	<u>0.998</u>	<u>0.999</u>	<u>0.999</u>
-75%	0.149	0.737	<u>0.915</u>	<u>0.969</u>	<u>0.988</u>	<u>0.996</u>	<u>0.998</u>	<u>0.999</u>	<u>0.999</u>	<u>0.999</u>
-80%	0.178	<u>0.806</u>	<u>0.945</u>	<u>0.982</u>	<u>0.994</u>	<u>0.998</u>	<u>0.999</u>	<u>0.999</u>	<u>0.999</u>	<u>1.000</u>
-85%	0.214	<u>0.858</u>	<u>0.964</u>	<u>0.990</u>	<u>0.997</u>	<u>0.999</u>	<u>0.999</u>	<u>0.999</u>	<u>1.000</u>	<u>1.000</u>
-90%	0.257	<u>0.896</u>	<u>0.976</u>	<u>0.994</u>	<u>0.998</u>	<u>0.999</u>	<u>0.999</u>	<u>1.000</u>	<u>1.000</u>	<u>1.000</u>
